# The role of environmental salinity on Na^+^-dependent intestinal amino acid uptake in rainbow trout (*Oncorhynchus mykiss*)

**DOI:** 10.1038/s41598-022-26904-6

**Published:** 2022-12-23

**Authors:** Ida Hedén, Kristina Sundell, Elisabeth Jönsson, Henrik Sundh

**Affiliations:** The Department of Biological and Environmental Sciences and SWEMARC (Swedish Mariculture Research Centre), The University of Gothenburg, Medicinaregatan 18A, 413 90 Gothenburg, Sweden

**Keywords:** Biochemistry, Physiology

## Abstract

Na^+^/K^+^-ATPases (NKA) in the basolateral membrane of the intestinal enterocytes create a Na^+^-gradient that drives both ion-coupled fluid uptake and nutrient transport. Being dependent on the same gradient as well as on the environmental salinity, these processes have the potential to affect each other. In salmonids, L-lysine absorption has been shown to be higher in freshwater (FW) than in seawater (SW) acclimated fish. Using electrophysiology (Ussing chamber technique), the aim was to explore if the decrease in L-lysine transport was due to allocation of the Na^+^-gradient towards ion-driven fluid uptake in SW, at the cost of amino acid transport. Intestinal NKA activity was higher in SW compared to FW fish. Exposure to ouabain, an inhibitor of NKA, decreased L-lysine transport. However, exposure to bumetanide and hydrochlorothiazide, inhibitors of Na^+^, K^+^, 2Cl^−^-co-transporter (NKCC) and Na^+^, Cl^−^-co-transporter (NCC) respectively, did not affect the rate of intestinal L-lysine transport. In conclusion, L-lysine transport is Na^+^-dependent in rainbow trout and the NKA activity and thus the available Na^+^-gradient increases after SW acclimation. This increased Na^+^-gradient is most likely directed towards osmoregulation, as amino acid transport is not compromised in SW acclimated fish.

## Introduction

The fish intestine is a multifunctional organ, absorbing nutrients for growth and metabolism, while also essential for osmoregulation to maintain homeostasis^1–5^. When anadromous salmonids move from freshwater (FW) to seawater (SW), they increase their drinking rate and the intestine performs ion-driven fluid absorption to replace fluid lost to the hyperosmotic environment^6–8^. Intestinal fluid absorption is tightly linked to the active uptake of Na^+^ and Cl^−^^9–11^. Two important apical co-transporters for Na^+^ and Cl^−^ uptake are the Na^+^, K^+^, 2Cl^−^ co-transporter (NKCC) and the Na^+^, Cl^−^- co-transporter (NCC), both of which are driven by the Na^+^ gradient^8,12–16^ created by the Na^+^, K^+^-ATPase (NKA) located on the basolateral membrane of the enterocytes^5,13,17^.

Inhibition of intestinal Na^+^ and fluid (J_v_) transport using furosemide and bumetanide as well as observations of NKCC2 mRNA expression, support the presence of NKCC in the intestinal tissue of both FW and SW acclimatized Atlantic salmon (*Salmo salar*), FW rainbow trout (*Oncorhynchus mykiss*), as well as in a range of other fish species^5,18–23^. The presence of NCC in the intestine of fish is also supported by a number of studies using either treatment with the specific NCC inhibitor hydrochlorothiazide (HCTZ), or by expression of NCC mRNA^19,20,24–26^.

In mammals, nutrient absorption, including glucose and several amino acids such as the essential amino acid L-lysine, is highly dependent on Na^+^-symport^27,28^. A number of Na^+^-driven transporters for nutrients (glucose and amino acids) have been identified in fish^29–39^. L-lysine is an essential amino acid limiting somatic growth in salmonids^38,40,41^. This amino acid shows amongst the highest absorption rates in the intestine, along with other essential amino acids such as methionine and proline^33,42^. In mammals, apical absorption of L-lysine is driven by the Na^+^-independent transporters rBAT (SLC3a1) and b^0,+^AT (SLC7a9), whereas the Na^+^-dependent neutral amino acid transporter b^0,+^AT1 (SLC6A19) shows low affinity for lysine^28,43^. Further, apical transport of L-lysine is coupled to basolateral y^+^LAT1 transporters that utilize Na^+^ or neutral amino acids as substrate to transport cationic amino acids across the membrane^43^. L-lysine is presumed to be absorbed through similar pathways in fish although this assumption should be made with caution since differences in amino acid transports has been seen^36,44^. This is supported by an upregulation in mRNA expression of rBAT and b^0,+^AT in the intestine of turbot fed higher concentrations of lysine^45^.

Intestinal osmoregulation is tightly linked to nutrient absorption at the biochemical level as both are driven by the Na^+^-gradient^46^. It is therefore probable that the activity in one system affects the other depending on the feeding and digestive status of the fish. Intestinal osmoregulation is considered more energetically costly in SW than in FW as indicated by increased intestinal blood perfusion and aerobic/anaerobic metabolism in parallel with elevated NKA activity after SW transfer in salmonids^47–53^. Previous studies on rainbow trout and Atlantic salmon show that the Na^+^-driven transport of glucose and the amino acids L-lysine, L-proline, is lower in SW compared to FW acclimated fish^54^. Further, net absorption of water and the monovalent ions, Na^+^ and Cl^−^, are shown to fluctuate considerably over time during digestion, and to differ between FW and SW acclimated rainbow trout^55,56^. In fact, Na^+^ and net water absorption were estimated to increase 5–25 times in SW acclimated rainbow trout during digestion^57^. In addition, the expression of Na^+^ dependent ion and nutrient transporters along with plasma amino acid concentrations, are altered depending on feeding status and environmental salinity^31,58–60^. It is unknown if the decrease in Na^+^-driven nutrient uptake in SW is a consequence of increased osmoregulatory load and thus, increased Na^+^-driven fluid uptake.

The aim of the current work was to examine the potential interaction between intestinal osmoregulation and nutrient transport in fed rainbow trout acclimated to FW and SW using L-lysine.

## Results

### Intestinal NKA activity and L-lysine transport in FW and SW

**Figure 1:**
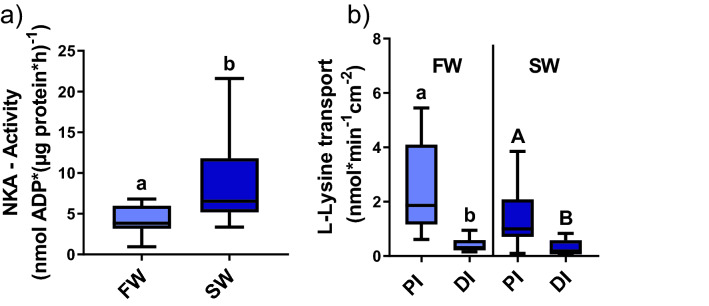
(**a**) NKA-activity in the proximal intestine of FW- and SW-acclimated rainbow trout. Different lowercase letters indicate significant difference in FW and SW acclimated rainbow trout (T-test; *P* = 0.02). (**b**) L-lysine transport. Different lowercase letters indicate significant difference in FW acclimated rainbow trout, and capital letter indicate significant difference in SW acclimated rainbow trout (T-test; FW *P* < 0.01; SW *P* = 0.01). Values are presented as min to max boxplots (n = 10–11).

Intestinal NKA activity was significantly increased in rainbow trout acclimated to SW in comparison to FW fish (T-test; *P* = 0.02; Fig. 1a). The L-lysine uptake was significantly higher in the proximal intestine than in the distal intestine in both FW (T-test; *P* < 0.01) and SW (T-test; *P* = 0.01) acclimated fish. Salinity did not affect L-lysine transport in any of the two intestinal regions (Fig. 1b). Tissue viability assessment using the paracellular diffusion marker D-mannitol (P_app_) did not show any significant differences between proximal or distal intestine in FW (9.9 × 10^−7^ ± 1.8 × 10^−7^; 6.6 × 10^−7^ ± 4.8 × 10^−8^ cm/s) and, SW (1.5 × 10^−6^ ± 4.3 × 10^−7^; 1.2 × 10^−6^ ± 4.3 × 10^−7^ cm/s). The proximal intestine was chosen for the detailed mechanistic studies as it was the main site of active L-lysine transport.

### Na/K-ATPase inhibition and L-lysine transport

**Figure 2:**
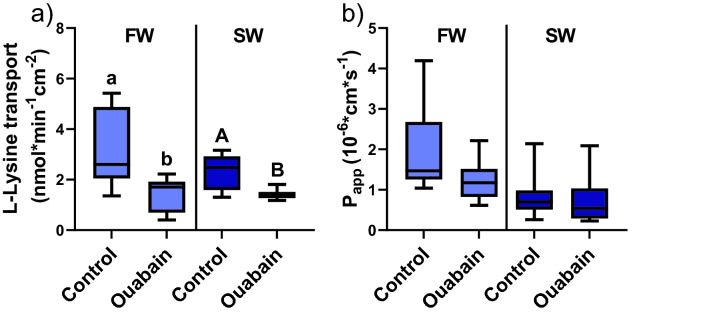
(**a**) L-Lysine transport across the proximal intestine of FW and SW acclimated rainbow trout in control or tissues exposed to ouabain for inhibition of NKA(T-test; FW *P* < 0.01; SW *P* < 0.01). (**b**) The apparent permeability coefficient of mannitol (Papp) in FW and SW acclimated rainbow in control and ouabain treated tissues for inhibition of NKA. Different lowercase letters indicate significant difference in FW acclimated rainbow trout, and capital letter indicate significant difference in SW acclimated rainbow trout. All values are presented as min to max boxplots and (n = 10–12).

**Figure 3:**
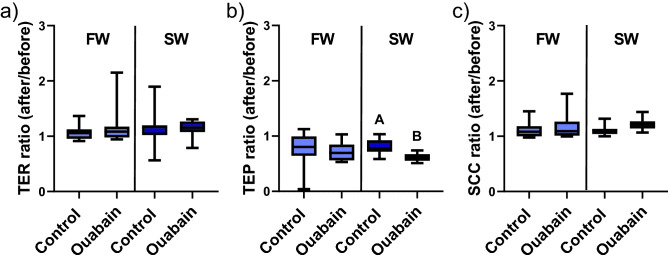
The ratio of the electrical parameters assessed in Ussing chambers (after/before) in response to 0.1% DMSO (control) or ouabain for (**a**) transepithelial electrical resistance (TER). (**b**) Transepithelial electrical potential (TEP). (**c**) Short-circuit current (SCC) in proximal intestines from FW and SW acclimated fish. All values are presented as min to max boxplots (n = 10–12).

Inhibition of NKA using ouabain decreased L-lysine transport across the proximal intestine in rainbow trout acclimated to both FW (T-test; *P* < 0.01), and SW (T-test; *P* < 0.01; Fig. 2a). The electrical viability assessment of the intestines from FW rainbow trout exposed to ouabain showed no significant effect of the inhibitors on Papp (Fig. 2b), TER, TEP, or SCC (Fig. 3a, b, and c). In SW there was a significant decrease in the intestinal ion transport activity indicated by TEP (T-test; *P* < 0.01; Fig. 3b), and a tendency towards a more negative SCC (T-test; *P* = 0.056; Fig. 3c). No effects of ouabain could be seen on the barrier functions in terms of Papp or TER.

### NKCC and NCC inhibition and L-lysine transport

**Figure 4:**
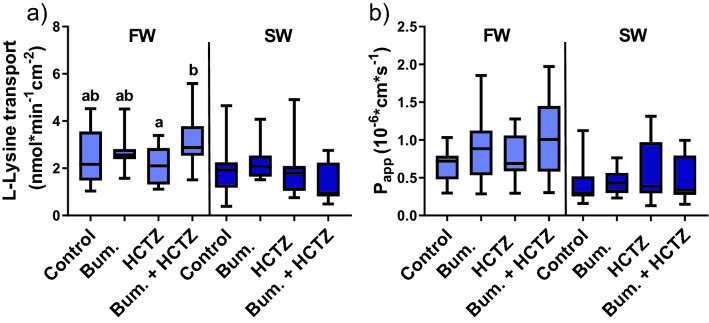
(**a**) L-lysine transport across the proximal intestine of FW and SW acclimated rainbow trout after exposure to control (0.1% DMSO), HCTZ, bumetanide and bumetanide + HCTZ simultaneously (Kruskal Wallis test; *P* = 0.05). (**b**) The apparent permeability coefficient (Papp) for mannitol across the proximal intestine of FW and SW acclimated rainbow trout after exposure to control (0.1% DMSO), HCTZ, bumetanide and bumetanide + HCTZ simultaneously. Different lowercase letters indicate significant difference in FW acclimated rainbow trout (pairwise comparison). All values are presented as min to max boxplots (n = 8–11).

**Figure 5:**
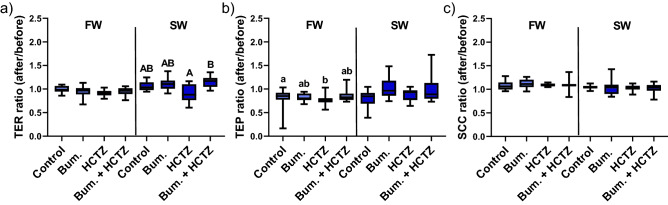
The ratio of the electrical parameters assessed in Ussing chambers (after/before) in response to 0.1% DMSO (control) or inhibitors (bumetanide, HCTZ and bumetanide + HCTZ) in the proximal intestine of FW and SW acclimated rainbow trout. (**a**) Transepithelial electrical resistance (TER) (Kruskal Wallis test; *P* = 0.01). (**b**) Transepithelial electrical potential (TEP) (Kruskal Wallis test; *P* = 0.02). (**c**) Short-circuit current (SCC). Different lowercase letters indicate significant difference in FW acclimated rainbow trout, and capital letter indicate significant difference in SW acclimated rainbow trout (pairwise comparison). All values are presented as min to max boxplots (n = 10–11).

Addition of inhibitors for the secondary active ion transporters NKCC/NCC, alone or in combination, to the mucosal side of the epithelia significantly affected L-lysine transport in FW (Kruskal–Wallis test; *P* = 0.05) but not in SW acclimated rainbow trout (Fig. 4a). In FW fish the combination of bumetanide and HCTZ resulted in significantly higher L-lysine transport in comparison to HCTZ alone (Pairwise comparison; *P* = 0.03; Fig. 4a). The inhibitors did not affect Papp in either FW or SW fish, indicating that the inhibitors did not affect the intestinal permeability for uncharged molecules (Fig. 4b). In SW acclimated fish TER was higher (Kruskal–Wallis test; *P* = 0.01; Fig. 5a) in the group exposed to Bumetanide + HCTZ in comparison to only HCTZ (Pairwise comparison; *P* = 0.01). No effects of the inhibitors were observed on TER in FW acclimated fish. Further, the net-ion distribution (assessed as TEP) was affected by the inhibitors in FW (Kruskal–Wallis test; *P* = 0.02), and multiple comparison analysis revealed that this emanated from decreased TEP after HCTZ exposure compared to control (Pairwise comparison; *P* = 0.03; Fig. 5b). No effects were observed on TEP in SW fish. Net ion transport (SCC) was not affected in FW, or SW acclimated rainbow trout (Fig. 5c).

### Intestinal fluid uptake (J_v_)

The fluid transport (J_v_) of FW acclimated fish was not significantly affected by bumetanide (8.9 ± 0.9 µl cm^−2^ h^−2^) or HCTZ (6.9 ± 0.8 µl cm^−2^ h^−2^) in comparison to the control exposed to DMSO (6.3 ± 0.9 µl cm^−2^ h^−2^) (One-way ANOVA).

## Discussion

There is a general agreement that the energetic cost for osmoregulation increases with increased deviation in osmolality between the external and internal environment^53,61^. The observed increase in NKA activity after SW acclimation supports previous findings that the energetic cost of intestinal transport activities increases in SW^48,52,62^. Further, the present study shows that at least 47% and 65% of the intestinal L-lysine uptake in rainbow trout is Na^+^-dependent and driven by NKA in FW and SW, respectively. Other amino acids that previously have been shown to be Na^+^-dependent in rainbow trout are leucine (Leu), methionine (Met) and valine (Val)^39^. In other fish species, lysine uptake, as well as that of several other amino acids (alanine (Ala), glycine (Gly), proline (Pro), aminoisobutyric acid (met-AIB), arginine (Arg), phenylalanine (Phe), α-amino-carboxylic acids, and taurine (Tau)), is Na^+^-dependent and affected by luminal Na^+^ concentrations^36,37,63–66^. This is in agreement with the prevalent mammalian model suggesting that intestinal amino acid transport is well preserved^28^. However, despite the increase in NKA activity in SW acclimated fish, Na^+^-driven L-lysine transport did not increase after acclimation to SW. This indicates that the increased Na^+^-gradient provided by the upregulation of NKA is directed towards the increased ion coupled fluid uptake needed in SW. Several other studies have demonstrated the presence of a reduced transport of several essential amino acids as well as glucose in SW acclimated compared to FW acclimated salmonids^33,42,67^. The present results, contrasting these previous studies and suggest that fully SW acclimated rainbow trout (> 4 weeks of SW acclimation) have the capacity to maintain the L-lysine transport in concordance with the increased solute linked water-transport. The ability to sustain amino acid transport in SW can be related to the osmoregulatory ability and/or timing of transfer of smolts. Coho salmon transferred to SW outside their smoltification window result in stunting, with reduced Na^+^-driven proline transport as a result. Whereas, fish transferred within the smoltification window sustain growth and show higher proline transport^68^.

The current study support that NKA activity is upregulated in SW concurrent with equal rates of Na^+^ driven L-lysine transport in both FW and SW. In contrast to previous studies reporting decreased Na^+^ driven nutrient transport in SW^33,42,68^. The inhibition of apical NKCC and NCC, the two co-transporters involved in ion-coupled water uptake, was therefore used to reduce the Na^+^ gradient used for ion-transport to be allocated to Na^+^-driven L-lysine uptake. The apical NKCC and NCC were inhibited using bumetanide, HCTZ or bumetanide and HCTZ together at concentrations previously shown to inhibit Na^+^ and/or fluid transport in both FW and SW acclimated fish^21,22,25,26,69,70^. However, in the current study the inhibitors had no cohesive effect on the electrophysiological parameters, nor did they decrease water transport using the gut sac methodology. Thus, under the assumption that NKCC and NCC were successfully blocked, the present study could not provide any evidence for an allocation of the Na^+^ gradient from ion transport towards Na^+^-driven lysine uptake. The decreased mucosa to serosa transport of nutrients including L-lysine previously demonstrated in SW acclimated fish^33,42,68^ is therefore likely a result of other unknown factors.

One probable explanation is that even if the NKCC and NCC were completely inhibited, the present amino acid transporters were already running at their full capacity and could therefore not be further increased. Traditionally examinations of osmoregulatory transport have been performed in starved fish, whereas later studies have shown that the transport of ions and water changes considerably in fed conditions^6^. In the current study, there were indications of low contribution of apical NKCC and NCC to net ion transport in fish in active feeding status. The lack of response from NKCC and NCC in continues presence of high nutrient concentrations does not necessarily indicate an impairment of the intestine to perform ion-driven fluid uptake. Contradictory, there is probably a difference in the importance of the different mechanisms of ion transport across the epithelium related to feeding status. During conditions when the intestinal lumen contains high concentrations of nutrients, Na^+^, Cl^−^ and thus fluid uptake may be driven, to a larger extent, by the ion transfer systems involved in nutrient absorptive and digestive processes. This suggestion is supported by the 5–25 times increase in the net absorption rate of Na^+^, Cl^−^ and fluid, in the proximal intestine of rainbow trout during digestion in SW acclimated fish^55,57^. During periods of fasting when the luminal nutrient concentration is minimal the apical NKCC and/or NCC are probably essential to sustain the ion-driven fluid uptake and hence osmoregulation.

In vivo, the active part of the transepithelial uptake of nutrients, including L-lysine, is a sum of uptake across the apical membrane, transfer across the basolateral membrane as well as transport and metabolism of the amino acids inside the enterocytes. Thus, a reduced transepithelial transport of amino acids seen in previous studies may also be a result of increased epithelial metabolism of amino acids by the enterocytes, in combination with or instead of a decrease in the active transport across the apical membrane. In mammals, enterocytes are known to mainly utilise amino acids as energy substrates for their metabolism, and they metabolize ~ 30–50% of ingested L-lysine^71–73^. In support, free amino acids concentrations in the intestinal mucosa were higher in SW acclimated rainbow trout in comparison to FW acclimated fish^74,75^. Further, in rainbow trout, high levels of ammonia and urea in the hepatic portal vein have been observed, suggesting that a large portion of the free amino acids taken up by the enterocytes is metabolised in the intestine^76^. This implies that reduced transepithelial nutrient transport in SW could be a result of higher metabolic consumption of amino acids in the intestinal tissue.

Collectively, the present and previous findings illustrate a dynamic intestinal transport system where several functions provide dual roles in maintaining osmotic and nutritional balance in both FW and SW acclimated fish. Future studies are warranted to explore the potential involvement of other transporters than those investigated in the present study. Uptake of all transported organic and inorganic solutes involved in the digestion and absorption of nutrients will create an osmotic gradient that drives fluid uptake also during feeding. It is also expected that a large portion of L-lysine and other nutrients will be catabolised in the enterocytes of the intestinal tissue during the process. From the whole animal perspective, the present study suggests that the Na^+^ driven nutrient transport for metabolism and ion transport for fluid uptake in the intestine of rainbow trout are not competitive, but complementary.

## Material and methods

### Experimental animals and holding conditions

Rainbow trout of both sexes were obtained from a local hatchery (Vänneåns fish farm AB, Knäred, Sweden) where they were reared in FW and transported to the experimental facilities at the Department of Biological and Environmental Sciences, Gothenburg, Sweden, at two different occasions. The first delivery of fish was received in summer 2015 (used for measurements of L-lysine uptake in proximal and distal intestine in FW and SW), and the second batch was received in early spring 2016 (used for inhibition of NKA, NKCC and NCC, see detailed description below). The mean start weight was 144 ± 38 g and the mean length were 21.6 ± 1.7 cm for both batches. Gut sac examination of water transport following exposure to bumetanide was performed on fish with a mean weight of 110.8 ± 6.1 g and length 21.8 ± 0.3 cm. The fish were kept in holding tanks in a recirculating FW aquaria system with a water temperature of 10˚C and a 12:12-h light–dark photoperiod. The fish were hand-fed ad libitum three times per week (4 mm Protec Trout pellets, Skretting, Stavanger, Norway). The experiment was started by transferring fish from the holding tanks into four identical tanks (1 m^3^) with either FW (salinity 0.1 ppt; two tanks), or artificial SW (salinity 30–33 ppt, sea salt Grotech, Ahorn, Germany; two tanks). The SW fish were allowed to acclimatize for a minimum of four weeks before sampling.

### Sampling

Each sampling day the fish were fed approximately two hours before sampling. The fish was euthanized in water containing MS-222 (200 mg l^−1^; Sigma-Aldrich, Stockholm, Sweden) buffered with HCO_3_^−^. The fish were then opened laterally, and the intestine was carefully rinsed from mesenteric fat using a tweezer. The proximal (from pyloric caeca to start of the distal intestine), and distal intestine (from the base of proximal intestine to anus) were sampled and opened longitudinally with a bull pointed scissor, and faeces was removed. The intestine was rinsed in modified ice cold FW or SW ringer solution (FW;140 mM NaCl; 2.5 mM KCl; 1.5 mM CaCl_2_; 0.8 mM MgSO_4_*7H_2_O; 15 mM NaHCO_3_; 1 mM KH_2_PO_4_; 5 mM HEPES; 0.5 mM L-lysine HCl; 10 mM D-Glucose; 20 mM L-Glutamine; pH 7.8), (SW; 150 mM NaCl; 2.5 mM KCl; 2.5 mM CaCl_2_; 1 mM MgCl_2_*6H_2_O; 7 mM NaHCO_3_; 0.7 mM NaH_2_PO_4_*2H_2_O; 5 mM HEPES; 0,5 mM L-lysine HCl; 10 mM D-Glucose; 20 mM L-Glutamine; pH 7.8)). For intestinal Na^+^/K^+^-ATPase measurements, mucosal tissue (~ 0.1 g wet weight) was scraped with the blunt side of a scalpel to separate it from the muscle and serosal tissue. The mucosal tissue was placed in ice-cold intestinal SEI buffer^48^ with protease inhibitors (2 μg mL^−1^ aprotinin, 1 mM PMSF) and 0.1% deoxycholic acid. The intestinal tissues were homogenized within a few hours after sampling (see below). The rest of the intestinal tissues was kept in ice-cold ringer solution for subsequent Ussing chamber experiments.

### Measurement of intestinal Na^+^/K^+^-ATPase

NKA activity was measured using a kinetic enzyme assay on fresh tissue^77^ modified for intestinal tissue^48^. In brief, mucosal tissue (~ 0.1 g wet weight) was homogenized with 20 strokes in a glass-glass homogeniser (Contes glass, Vineland, NJ, USA) on ice. The homogenate was centrifuged (3000 g at 4 °C for 3 min) and the supernatant was used for further analysis. The absorbance readings of NADH were performed in a microplate reader (SpectraMax 190, Molecular Devices Corp., Menlo Park, CA, USA) at 340 nm for 10 min at 25 °C. Specific NKA activity is expressed as nmol ADP × (µg protein × h)^−1^. The protein content of the sample was measured using the bicinchoninic acid (BCA) protein assay kit according to the manufacturer’s instructions (Pierce, Rockford, IL, USA).

### Ussing chamber

The Ussing chamber method used is previously described in detail by Sundell and Sundh^16^. In short, transepithelial resistance (TER), transepithelial potential (TEP), and short circuit current (SCC) were measured across the epithelia using modified Ussing chambers with 4 ml solutions on the mucosal and serosal side with a total of 0.75 cm^2^ exposure area. The tissue was aeriated using a gas mixture of 99.7% air and 0.3% CO_2_. The chambers were set in a water-filled cooling mantel with 10 °C circulating water. Measurements were performed with 5 min interval to avoid epithelial capacitance**.**

### L-lysine transport in proximal and distal intestine in FW and SW

The assessment of L-lysine transport in FW and SW was performed on proximal and distal intestinal regions from twelve individual fish (n = 12). The intestines were allowed to acclimate in the chamber in fresh Ringer’s solution for 30 min. After 30 min the ringer was exchanged in both chambers; the serosal side received new fresh Ringer`s solution and the mucosal side received Ringer’s solution containing ^3^H-L-lysine 17 µBq/µL (PerkinElmer, Waltham, MA, USA), and 0.5 mM unlabelled L-lysine for measurements of active L-lysine transport. Further, the paracellular marker molecule ^14^C-mannitol (17 µBq/µL; PerkinElmer, Waltham, MA, USA) was added to the mucosal side for assessment of tissue integrity. After an additional 30 min, measurement of accumulated radioactivity over 90 min was initiated by withdrawing 100 µL ringer from the mucosal and serosal chamber at T = 0. Thereafter 100 µL samples were taken from the serosal chamber at: T = 20, 30, 60, 80 and 90 min and exchanged for new Ringer. Five mL scintillation fluid (Ultima Gold, PerkinElmer, Waltham, MA, USA) was added to each 100-µL sample and radioactivity was determined using a β-counter (Wallac 1409 Liquid Scintillation Counter, Turku, Finland).

The mucosal to serosal L-lysine transport across the epithelium was calculated according to the equation:

$$L{ - }lysine\;transport = \frac{ dQ/dt}{A}$$

where dQ/dt is the accumulation of L-lysine over time (mol/min) divided by the intestinal tissue area (A = 0.75 cm^2^).

The apparent permeability coefficient (P_app_) for mannitol was calculated using the equation:

$$P_{app} = \frac{dQ}{{dt}}* \frac{1}{{AC_{0} }}$$

where dQ/dt is serosal accumulation of ^14^C-mannitol over time (mol/s) of and C_0_ is the initial concentration on the mucosal side (mol/ml) and exposed tissue area (A = 0.75 cm^2^). If the resistance of the tissue decreased or if P_app_ was extremely high this was interpreted as tissue damage and the sample was excluded before data analysis.

### Na/K-ATPase inhibition and L-lysine transport

The proximal intestine from 12 FW and SW fish was exposed to ouabain (0.1 µM final concentration; for inhibition of NKA; Sigma-Aldrich, Stockholm, Sweden). The Ussing chamber experiment followed the same protocol as previously described except for the addition of ouabain solubilised in 0.1% DMSO to the serosal half chamber and 0.1% DMSO to the mucosal half chamber at T = 30, when exchanging ringer. The control was exposed to 0.1% DMSO in both half chambers (n = 12).

### Effects of NKCC and NCC inhibition on electrophysiology

Sampling was performed as previously described on proximal intestine from FW and SW acclimated fish (n = 12). The Ussing chamber experiment followed the same protocol as previously described. The NKCC inhibitor Bumetanide (Sigma-Aldrich, Stockholm, Sweden), and NCC inhibitor hydrochlorothiazide (HCTZ; Sigma-Aldrich, Stockholm, Sweden) previously demonstrated to be effective in SW and FW acclimated rainbow trout were applied^22,26^. The two inhibitors were added separately or in combination, at T = 30, to the mucosal half chamber at final concentrations of 0.1 mM. The inhibitors were solubilised in 0.1% DMSO (Sigma-Aldrich, Stockholm, Sweden), and thus the serosal half chamber was exposed to 0.1% DMSO.

### Intestinal fluid uptake (J_v_)

Non-everted gut-sacs^62^ were used to examine the influence of HCTZ and bumetanide on water transport using gravimetric assessment. The proximal intestine from FW rainbow trout were tied at the distal end and filled with Ringer´s solution (as described for Ussing, but without addition of nutrients; 10 °C) containing 1 mM HCTZ (n = 8) or 1 mM bumetanide (n = 8), or 0,1% DMSO (n = 8) and tied at the proximal end. The sacs were incubated in 50 ml falcon tubes containing Ringer´s, aerated using a gas mixture of 99.7% air and 0.3% CO_2._ The tubes were kept in a cooling bath at 10 °C. Measurements were initiated by weighing the original sac and thereafter subsequent gentle blotting of the sac and weighing every 30 min for 2 h. The rate of water transport was determined using linear regression, normalized to the mucosal surface area. The net mucosal to serosal water transport is expressed as µl cm^−2^ h^−2^.

### Statistics

All data were assessed for normal distribution by visual inspection of histograms and QQ plots, as well as through Shapiro-Wilks’s tests. Homogeneity of variance was tested using Levene´s test of equal variances. If the data failed to meet the criteria for normal distribution or equal variances, it was transformed in the order of SQRT and then LG10. Obvious outliers due to epithelial damage in the Ussing chambers (drop in TER and/or sudden increase in Papp) were removed from the data set. When the data fulfilled all criteria for parametric testing, they were further assessed using independent T-tests or one-way ANOVA. If the data still failed the assumption of normal distribution or equal variances after transformation, they were analysed using a non-parametric Kruskal–Wallis test. Statistical differences were determined using pairwise comparisons with Bonferroni correction. All values are presented as min to max boxplots or as mean ± SEM. *P* values < 0.05 were considered to indicate statistical differences. Statistical analyses were performed using SPSS statistical software (Version 24, IBM Corp, Armonk, NY, USA).

### Ethics declaration

The experiments were approved by the regional ethical committee for animal experimentation (licence number 177-2013). The ethical regulations follow national and European ethical guidelines on animal experimentation under the EU directive 2010/63/EU, and the ARRIVE guidelines.

## Summary and conclusion

The obtained data show that L-lysine transport in rainbow trout is dependent on the Na^+^-gradient built up by NKA. However, increased NKA-activity in SW did not stimulate L-lysine transport. Instead, the increased Na^+^-gradient appears to be allocated to osmoregulatory processes. Further, this study could not find evidence for an apical location of either NKCC or NCC in FW or SW acclimated rainbow trout. This is likely linked to the active feeding status of the fish. The temporal changes in ion-transport and nutrient transport pathways in fed and unfed conditions warrants further investigation.

## Data Availability

All data generated and/or analysed during the current study are available upon request from the corresponding author.
